# Rheumatic Heart Valve Disease Pathophysiology and Underlying Mechanisms

**DOI:** 10.3389/fcvm.2020.612716

**Published:** 2021-01-18

**Authors:** Livia S. A. Passos, Maria Carmo P. Nunes, Elena Aikawa

**Affiliations:** ^1^The Center for Excellence in Vascular Biology, Harvard Medical School, Brigham and Women's Hospital, Boston, MA, United States; ^2^Hospital das Clínicas e Faculdade de Medicina da Universidade Federal de Minas Gerais, Belo Horizonte, Brazil; ^3^Department of Human Pathology, Sechenov First Moscow State Medical University, Moscow, Russia

**Keywords:** rheumatic heart disease, mitral valve, inflammation, autoimmunity, pathogenesis

## Abstract

Rheumatic heart valve disease (RHVD) is a post-infectious sequel of acute rheumatic fever resulting from an abnormal immune response to a streptococcal pharyngitis that triggers valvular damage. RHVD is the leading cause of cardiovascular death in children and young adults, mainly in women from low and middle-income countries. It is known that long-term inflammation and high degree of fibrosis leads to valve dysfunction due to anatomic disruption of the valve apparatus. However, since public and private investments in RHVD studies are practically inexistent the number of publications is scarce. This disease shows different natural history and clinical presentations as compared to other degenerative heart valve diseases. Although more than five decades passed after the pioneering studies on the pathogenesis of RHVD, it is still unclear how self-tolerance mechanisms fail in this disease, and how humoral and cellular inflammatory responses are interconnected. Despite that pathological mechanisms have been already proposed for RHVD, none of them are able to explain the preferential involvement of the mitral valve. This review focuses on pathophysiology and underlying mechanisms of RHVD.

## Introduction

### Rheumatic Heart Valve Disease

Heart valve disease (HVD) is generic term that includes several etiologic entities with different pathophysiologic mechanisms that lead to anatomic disruption of the valve apparatus (1). Functional abnormalities due to alteration in matrix architecture and cellular components impair the proper directionality of blood flow through the heart chambers resulting in heart failure (2).

Overall, HVD are slowly progressive disorders that affect mainly the aging population (>65 years), reaching epidemic proportions worldwide (3). Despite increased life expectancy over the last several decades, calcific aortic valve disease (CAVD), and degenerative mitral valve disease are the two most common types of non-rheumatic valve disease (4). Due to vast health impact in developed world, CAVD has sustained significant research interest and a greater number of studies as compared to the other valvular disorders.

On the other hand, in low and middle-income countries, rheumatic heart valve disease (RHVD) is the leading cause of cardiovascular death in children and young adults (5, 6). Even though the observed progress in research on RHVD pathogenesis with findings that have challenged a variety of historical paradigms, a number of key scientific questions remain. Public and private investments in RHVD studies are low and therefore the number of publications is limited. RHVD could be viewed as a marker of inequality and social injustice for countless populations living in poverty. Over the last years, defense groups are making efforts for identification and removal of barriers to the translation of existing knowledge into policy, programs, and practice to provide high-quality care for patients with RHVD (7).

HVD require a substantial allocation of health resources, and there is no effective drug therapy to prevent or treat these pathologies. In addition, these valvular diseases show different natural histories and clinical presentations. This review focuses on pathophysiology and underlying mechanisms of RHVD.

## Pathology of RHVD

### Gross Pathomorphological Findings

RHVD is a harmful post-infectious sequel of acute rheumatic fever (ARF) resulting from an abnormal immune response to a streptococcal pharyngitis that triggers valvular damage (8). The first episode of ARF is often associated with only mild manifestations and can occur at any age in genetically predisposed individuals (9). Recurrent *Streptococcus pyogenes* infection, which boosts immune response leads to RHVD. Thus, although RHVD first occurs in childhood, its incidence peaks in adulthood, usually between the ages of 25–45 years (10). In developing countries, social determinants of the disease such as inadequate housing, lack of access to primary health care, education, and availability of cardiologic diagnostic tools hamper the diagnosis. Most of children are undiagnosed and therefore do not receive antibiotic as secondary prophylaxis to prevent new *S. pyogenes* increasing their chances to develop RHVD. Women and girls may experience less access to primary and secondary prophylaxis as compared with men and boys in low-income countries, and this could also contribute to differences in RHVD rates between females and males (9). In addition, women have a closer involvement in childcare and therefore higher *S. pyogenes* exposure.

**Figure 1:**
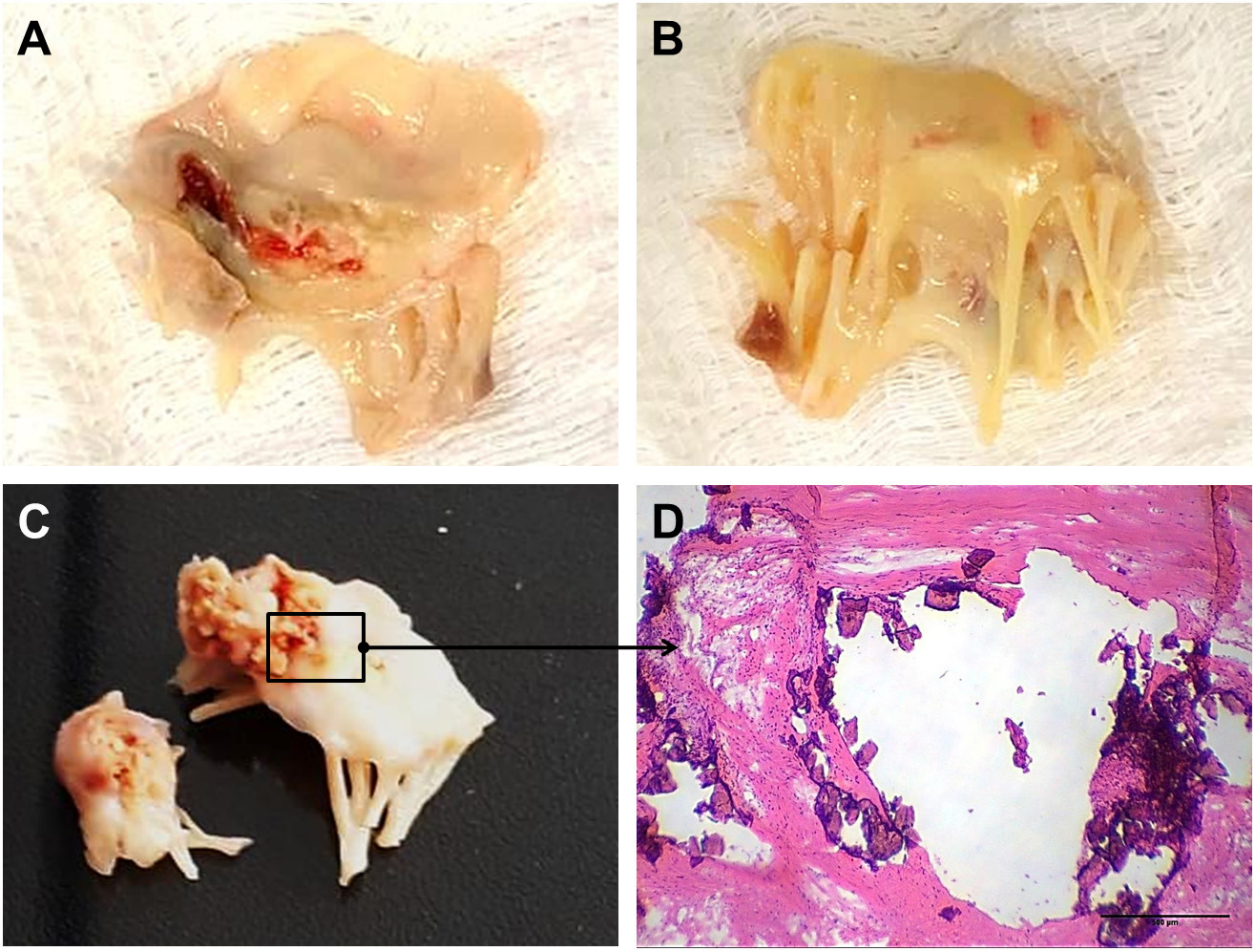
Gross pathology and histological aspects of rheumatic mitral valve at the end stage of RHVD. **(A,B)** Atrial and ventricular sides of mitral valves excised from female, 49 year-old patient, showing thick leaflets with retraction. **(C)** Mitral valve excised from male, 61 year-old, showing calcification. **(D)** Representative Hematoxylin and Eosin staining of anterior mitral valve leaflet showing presence of nodular calcification. Scale bar = 500 μm.

**Figure 2:**
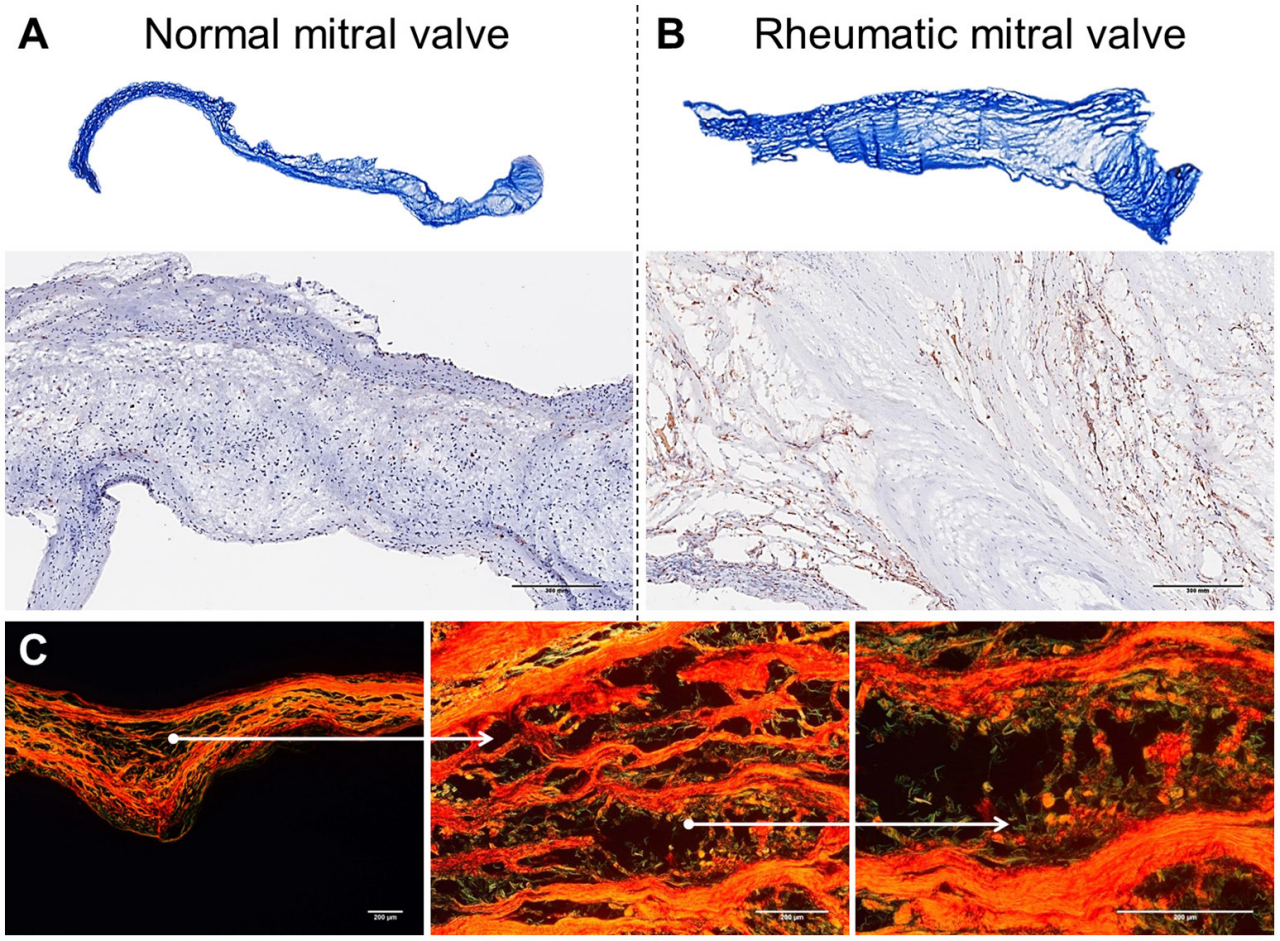
Histological comparison between normal mitral valves and rheumatic mitral valves. **(A)** Masson trichrome staining showing focal fibrosis and thickening of rheumatic mitral valves. Representative images are shown. **(B)** Representative immunohistochemistry staining for CD45^+^ cells evidencing a high frequency of leukocytes. Scale bars = 300 mm. **(C)** Picrosirius red staining showing uneven collagen deposition. Representative images are shown. Scale bars = 200 μm.

The mitral valve is affected in almost all RHVD cases, with regurgitation in the early stages, and stenosis in later stages (11). RHVD can also affect aortic valves, however, calcific degeneration is an outcome usually associated with aortic valve. During initial phase of rheumatic disease, echocardiographic exams can detect small verrucous nodules caused by the presence of thrombi along the lines of heart valve closure. These lesions are not able to produce leaflet destruction and therefore valve function is relatively normal. On the other hand, development of long-term inflammation after single or multiple episodes of rheumatic fever can lead to valve dysfunction in untreated genetically predisposed patients. As general pathomorphological findings, mitral valve specimens from patients at end state disease are thick and stiff due to a high degree of fibrosis (Figures 1A,B). As this process stretches over decades, different morphological changes dominate the various phases. While leaflets are usually minimally fibrose and pliable in three quarters of patients younger than 30 years of age, they are scared and ridged in two thirds of patients older than 40 years. Different morphological manifestations also lead to different clinical symptoms. While chordal shortening is dominant in 90% of patients with mitral stenosis, it only occurs in 3% of patients with pure mitral regurgitation (MR). Annular dilatation is found in 90% of patients with pure MR but only in 30% of cases with pure stenosis. In most late cases, the valve commissures are fused and often endothelium surface erosion is observed. Chordae tendineae show fusion and shortening (Figure 1B), which may reduce subvalvular chordal space (12, 13). Calcification occurs in some cases of RHVD (Figures 1C,D), however fibrosis and inflammation are major findings (Figures 2A–C) (14, 15). Accumulating studies had shown that lipids may trigger vascular calcification associated with atherosclerosis. Therefore, it is possible that RHVD patients who develop valve calcification have altered cholesterol profile. It still remains a gap of knowledge in RHVD research.

## Mechanisms of Mitral Valve Disease

### Immune Response in RHVD

Mitral valves are populated by two major types of cells: valvular endothelial cells (VEC), covering the leaflets, on both atrial and ventricular sites; and valvular interstitial cells (VIC), quiescent fibroblast-like cells, which are important in the homeostatic remodeling of matrix constituents (16–19). In disease state, cell composition shifts toward contractile and collagen-producing myofibroblast-like cells leading to fibrotic changes and stiffness of leaflets (Figure 2A) (19). Chronic inflammatory processes are dominant in RHVD resulting in accelerated loss of valve function (Figure 2B). Mitral valve is composed of three layers of specialized connective tissue between the two endothelial layers and its architecture substantially changes during RHVD progression. Anatomical features of mitral valve can be associated with its preferential involvement in this disorder. However, the underlying mechanisms of mitral valve predominant involvement in RHVD are unknown.

Studies related to immunopathogenesis of rheumatic fever as well as of the RHVD have been conducted since 1960 when the presence of autoantibodies in the serum of patients with throat infection by group A β-hemolytic Streptococci has been demonstrated (20). Although more than five decades passed after the pioneering studies on the pathogenesis of RHVD, little progress has been made in respect of the cellular and molecular aspects, which lead to the destruction of the valve tissue. In RHVD patients, the generation of an antibacterial immune response starts in the pharyngeal epithelium by innate immune components such as neutrophils, macrophages, and dendritic cells. These cells recognize and process bacterial antigens and present them to B lymphocytes, culminating in the production of immunoglobulins that are able to recognize epitopes in several host sites and activate T cells.

Still a mystery the mechanisms involved in the loss of self-tolerance in RHVD and how immune system target heart valvular tissue, especially mitral valve. Autoimmune valvular carditis are mainly described in literature as associated with human rheumatic conditions, however, it is possible that autoreactive antibodies can be associated with pathology of other HVD, including CAVD. Ectopic calcification and autoimmunity has been also explored in atherosclerosis (21, 22).

Multiple bacterial antigens are involved in RHVD damage. M, T and R proteins and N-acetylglucosamine (GlcNac), a group A β-hemolytic Streptococci carbohydrate (GAS), are the main epitopes described to be associated with molecular mimicry. These molecules share structural similarity with host cardiac myosin, laminin, vimentin, and tropomyosin. Many studies point to M protein as the most virulent protein (23, 24). Although myosin is present in the myocardium but not in the valvular tissue, anti-myosin antibodies respond against GlcNac epitope due to similar structures of common alpha-helical sequences and glycosylated proteins (25). It is assumed that myosin is an intracellular protein and therefore immunologically inaccessible, and thus does not participate as the initial target of cross-reactive antibodies. However, after initial mild endothelial breakdown, intracellular epitopes may contribute to robust amplification of the immune response due to increased availability of binding sites for anti-myosin antibodies.

Investigations have highlighted an important participation of GlcNac antigens in RHVD pathogenesis, since persistent levels of anti-GAS antibodies were correlated with valvulitis. It is also observed that after valve replacement the serum levels of anti-GAS were reduced (23, 26). In addition, unlike to some of the cross-reactive intracellular proteins, GlcNac are cell-surface antigens that are more exposed/accessible to antibody recognition (27). In non-rheumatic valve disease, the triggering factors leading to cardiovascular calcification, as in CAVD are still under investigation (22).

Although pan-carditis occurs in the early disease stages, it is reversible, and only the valvular damage is permanent, especially in mitral valve. Mitral valve damage is initiated by circulating autoantibodies that bind to the endothelial surface of the valves, leading to increased expression of vascular cell adhesion protein 1 (VCAM-1). The activated endothelium facilitates the infiltration of T lymphocytes into the valvular subendothelium at the endocardium site, leading to edema and elongation of the chordae tendinae (28, 29). Due to tissue injury, components of ECM are exposed and anti-collagen antibodies are produced. These antibodies can deposit in the valve contributing to pro-inflammatory environment. All these changes cause the heart valves to be a vulnerable immune-privileged site for injury. The role of anti-collagen antibodies will be addressed in more details below.

It is important to comment that typically the first streptococcus throat infection does not trigger an episode of rheumatic fever. Recent studies have shown that continuous infections maintain the germinal center reaction and affinity for antibody maturation (30, 31). As such, preexisting immune complexes would capture more immunoglobulin leading to amplification of the immune response, which further favors the recognition of several self-antigens and propagate tissue damage. Thus, repeated infections feed the disease onset (32). To date, no evidence exists that the isolated presence of valve-reactive antibodies in the serum of RHVD patients is sufficient to produce the valve lesion, suggesting the importance of cellular response besides humoral components. In addition, autoantibodies are often found in patients after uncomplicated streptococcal pharyngitis and in healthy individuals.

Humoral and cellular response acts together in autoimmune diseases. It is known that women produce more immunoglobulin than men, and the X chromosome contains over 1,000 genes, while the Y chromosome only has about a 100 (33). Many of the X-linked genes are relate to the immune system, such as CD40L, CXCR, OGT, FOXP3, TLR7, TLR8, IL2RG, BTK, and IL9R. Also, sex hormones can directly or indirectly affect the immune response by modulating gene expression through ERα stimulation (34). Immune cells express estrogen and androgen receptors, and engagement of these receptors affects lymphocyte responses (35). ARF usually occurs during childhood equally in males and females, however, RHVD has higher prevalence in adult women. Thus, is likely that endogenous hormones are key mediators of disease progression. Overexpressed X-linked immune genes and estradiol probably act in a synergistic manner, leading to a greater female-biased predisposition in RHVD.

Emerging evidence suggests that the transfer of T-cell lines from M-protein vaccinated Lewis rats to naïve animals can induce valvulitis in recipient animals. These data support that T cells are sufficient to induce inflammation, not requiring the presence of cross-reactive antibodies to trigger valvulitis (36). Although some studies showed that the presence of antibodies is not crucial in triggering RHVD pathogenesis, it is important to emphasize that the ability of antibodies to become self-reactive will depend on the combination of factors such as genetic background, recurrence of infections, and strain virulence. These variables make it even more challenging to fully understand the mechanisms associated with the development of valve lesions.

**Figure 3:**
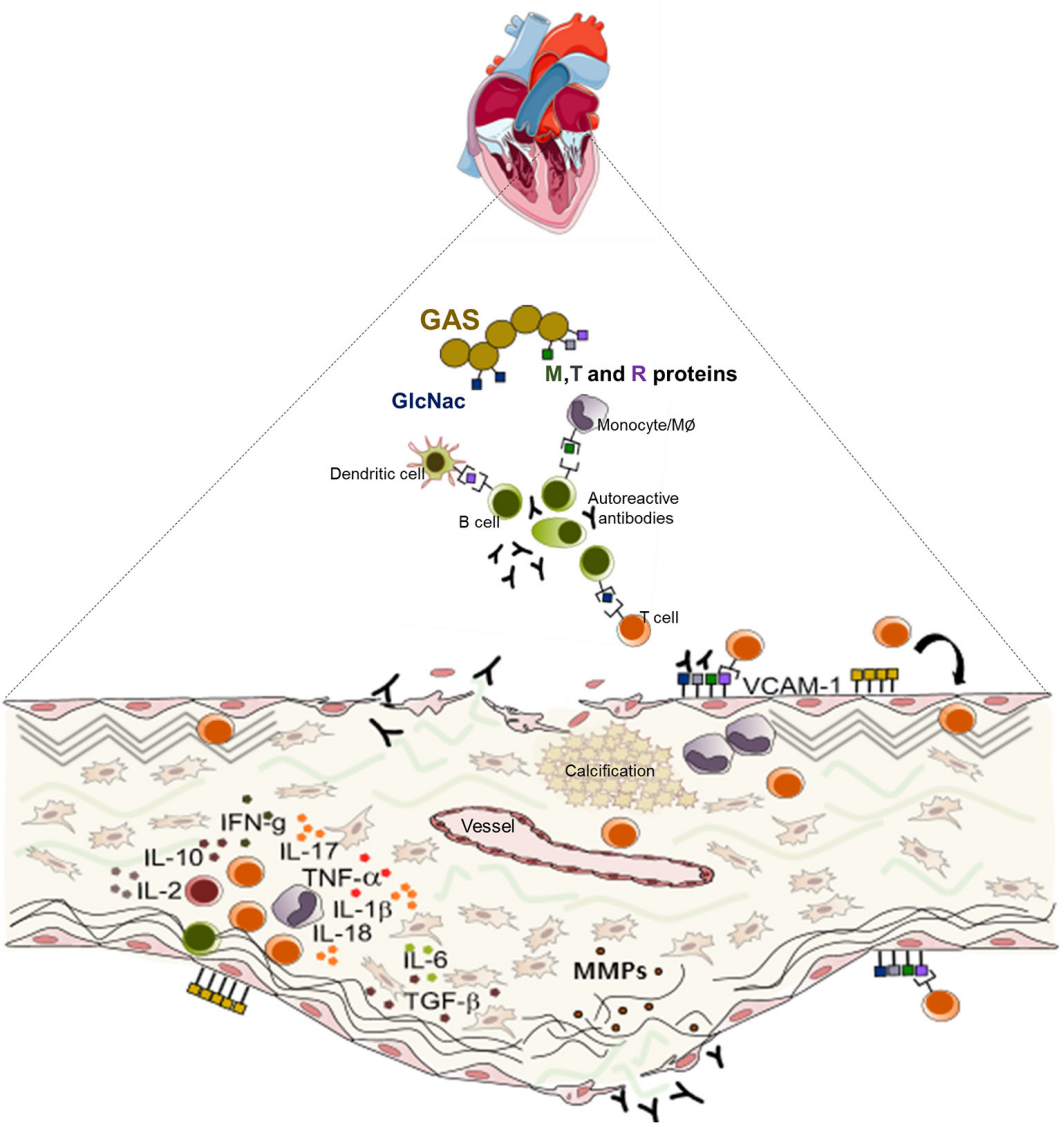
Schematic representation of the mechanisms of the pathogenesis of rheumatic heart valve disease. Following group A Streptococcus (GAS) invasion of the pharyngeal epithelium, GAS recognize and process bacterial antigens and present them to B lymphocytes. Activated B cells produce antibodies that are able to recognize epitopes in several sites in the host and also activate T lymphocytes. In the heart, cross reactive T cell clones and antibodies act against heart valve constituents leading to an intense inflammatory process culminating in valve dysfunction.

The inflammatory infiltrate described in the rheumatic mitral valve of patients in the end state of disease are predominantly composed of mononuclear cells (Figure 3C), mainly helper (Th) - CD4, and cytotoxic—T CD8 lymphocytes, macrophages and B cells (37, 38). The effector function and therefore the contribution of these cells in the pathogenesis of RHVD is largely associated with the profile of cytokines and other soluble mediators produced by them that lead to differentiation of VICs to activated collagen-producing myofibroblast (39).

So far, T CD4^+^ lymphocytes have been the most investigated cell population in RHVD since they are present at high frequency in the inflammatory infiltrates and have high cross reactivity against cardiac myosin epitopes. In addition, these cells are able to differentiate into various repertoires of subpopulations producing diverse cytokines leading to the development of different degrees of cross-reactivity (40–43).

Th1 cytokines are pro-inflammatory soluble mediators involved in host defense and also crucial to autoimmunity. In the RHVD context, some studies have evaluated the expression of different inflammatory cytokines in the valvular tissue, however cell sources of these cytokines and contributions of each cellular subpopulation to the cytokine production remain unknown.

Among the Th1 cytokines, TNF-α, IFN-γ, IL-1, IL-2, and IL-6 have already been shown to be associated with disease progression. *In vitro* studies have demonstrated that TNF-α exhibits a high chemotactic potential promoting cell attraction to the site of inflammation, whereas IFN-γ can induce processing and presentation of autoantigens (38, 44, 45). IL-1 is a key cytokine in autoimmune disease and has also been shown to be associated with inflammatory damage, especially in the acute phase of rheumatic disease (46, 47). In a Brazilian population study, polymorphism in IL-Ra and IL-6 genes was associated with susceptibility to RHVD (48, 49). IL-6 was linked to B cell antibody production and suggested to be involved in RHVD pathogenesis (50). Systemic cytokine levels were associated with severity of RHVD and co-regulated expression of IL-6 and TNF-α was associated with severe valve dysfunction (51).

IL-2 is an essential cytokine initiating generation of regulatory T cells (Tregs), which play a vital role in the maintenance of immune tolerance. Low levels of IL-2 and deficiency of circulating Tregs were associated with rheumatic mitral valve disease (44, 52, 53). Moreover, patients who presented multiple valve impairment showed a greater deficiency in the number of Tregs (54).

Th17 cells are T CD4^+^ cells producing high amounts of IL-17. This cell subset plays opposite roles as compared to Tregs in autoimmune diseases. In the context of RHVD, it was shown to be associated with progression of the disease toward chronic state in an experimental model (55). Peripheral blood cells from patients with rheumatic mitral valve disease showed an increased number of Th17 cells and high serum levels of IL-17 as compared to healthy individuals (52).

Some cytokines with a typical Th2 response mediating activation and regulation responses against allergen toxins, extracellular parasites and bacteria, have also been studied in RHVD. In these patients, IL-10 is present at high levels and has a direct correlation with T CD8^+^ lymphocyte response. IL-10 acts as a chemoattractant for these cells and creates a favorable milieu for the growth of its precursors. On the other hand, Th2 cytokines such as IL-4 and IL-5 are present in very low concentrations or not detectable in RHVD (56–58).

The role of cytokines in the site of inflammation is still underexplored since the vast majority of studies were done using peripheral blood, and the expression of cytokines at the lesion site could be underestimated. Analysis of valve tissue is essential for the understanding of inflammatory mechanisms *in situ*. Studies performed in peripheral blood and valvular tissues from RHVD patients, point to a predominance of T CD4^+^ cells as compared to the number of T CD8^+^ cells. However, the proportion of circulating T CD4^+^ and T CD8^+^ cells appears to vary between stages of the disease, as evidenced by increased frequency of T CD8^+^ cells in patients with chronic stage disease (37, 38, 44). The role of T CD8^+^ cells in the pathogenesis of RHVD, however, remains scant.

B lymphocytes are present in the inflammatory infiltrates of rheumatic mitral valves. While their contribution to the pathogenesis of the disease is often associated with the production of antibodies in the early disease stages, it is likely that these cells also play a role of effector cells participating in chronic lesion development (38, 59).

Macrophages are known to play a high pathogenic role in cardiovascular diseases. A recent study showed that pro-inflammatory macrophages (M1) exhibit effector potential through activation of NLRP3 inflammasome (60) leading to the production of IL-1β and IL-18, an important pathway in the pathogenesis of rheumatic diseases (61, 62). IL-1β in turn, induces release of matrix metalloproteinases (MMPs), recruitment and proliferation of resident fibroblasts, and secretion of TGF-β and IL-6 resulting in the development of fibrosis (63).

### Collagen Remodeling and Calcification Process in RHVD

More recent studies have proposed that the immune response in RHVD may not be merely related to molecular mimicry or failure of the immune system, but rather associated with collagen autoimmunity as proposed for Goodpasture and Alport syndromes (64). In these diseases, the production of autoantibodies against basement-membrane collagen (type IV) on host endothelium is the triggering step of pathological processes. In Streptococci infection, M protein binds to CB3 region of collagen IV leading to the formation of a complex that promotes conformational changes in collagen structure initiating an anti-collagen response (65–67). Thus, a ubiquitous protein can become a self-antigen that contributes to imbalance between collagen deposition and collagen degradation, culminating in subsequent fibrosis of the valve apparatus in the RHVD.

Mitral valves of rheumatic patients have a higher deposition of collagens Type I and Type III, evidence of fibrosis, when compared to non-rheumatic mitral valve controls (Figures 2A–C) (68). Among numerous cytokines involved in the inflammatory process in RHVD, the high expression of TGF-β has been shown to be positively associated with valvular fibrosis (69) by contributing to myofibroblast activation and collagen production (39).

MMPs are a major group of proteases that regulate matrix remodeling during fibrogenic process accompanying chronic inflammation. MMP-1 has a high affinity to fibrillar collagens and is able to initiate collagenolysis. High concentrations of MMP-1 in patients' plasma, and gene polymorphism are contributing risk factors for RHVD (68, 70). The majorities of studies addressing the role of MMPs were performed in human plasma and myocardium tissues during acute episodes of rheumatic fever and are scarce in the chronic disease phase, particularly in valves (71–73).

Proinflammatory MMPs also play a role in the modulation of calcification by elastin degradation. Exposure of elastin and matrix-bound cytokine generation after tissue injury create a milieu, which promotes the smooth muscle cell changes into osteoblastic phenotype (74, 75). Calcification is a very common finding in rheumatic mitral valves, however, the cellular mechanisms responsible for the calcification in RHVD are not well-understood (Figure 3B). Previous study reported that mineralization occurs in areas of inflammation and neoangiogenesis, which express vascular endothelial growth factor (VEGF) (15). This molecule is able to regulate bone remodeling by attracting endothelial cells and by stimulating osteoblast differentiation (76). Another potential mechanism involved in mitral valve mineralization in RHVD is through calcification-competent extracellular vesicles derived from smooth muscle cells, VICs or macrophages (77–79). Calcification in RHVD seems to be triggered by inflammatory process as observed in CAVD (2, 22).

Together, the immune response triggered by pharyngeal GAS infection results in a cascade of cellular and humoral events that culminate in the production of antibodies and generate self-reactive clones of lymphocytes capable of interacting with valve components leading to leaflet tissue degeneration. Schematic presentation of the mechanisms of RHVD pathogenesis is shown in Figure 3.

### Neoangiogenesis in RHVD

**Figure 4:**
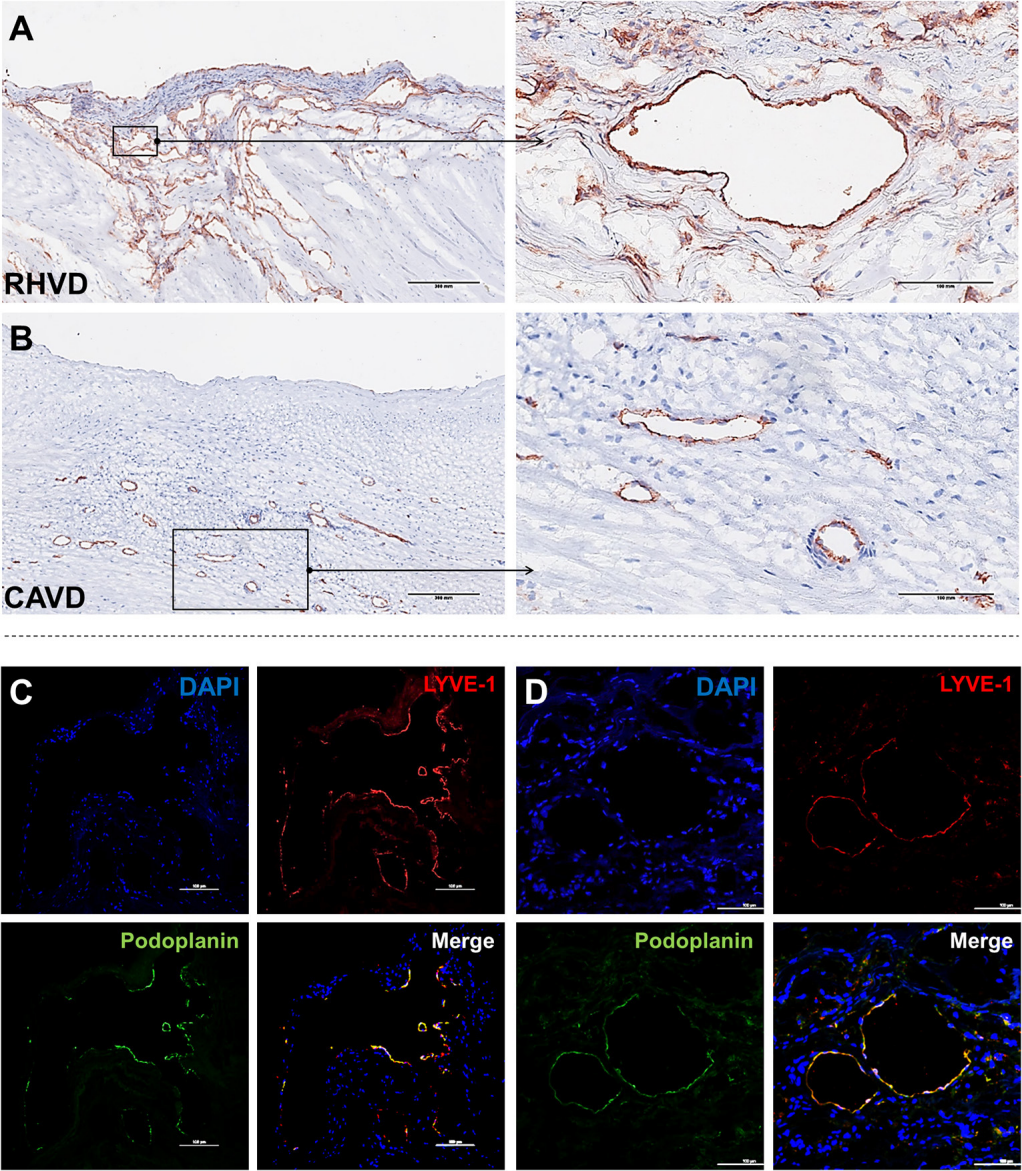
Histological comparison between neoangiogenesis in rheumatic mitral valve and calcific aortic valve. **(A,B)** Representative immunohistochemistry image for CD31^+^ staining evidencing presence of immature vessels in RHVD mitral valve and calcific aortic valve interstitium. Scale bars = 300 mm. **(C)** Representative immunofluorescence image showing cell co-expressing LYVE-1 and podoplanin demonstrating presence of lymphatic vessels in rheumatic mitral valves. Scale bars = 100 μm.

Neoangiogenesis is a common feature of HVD acting as a facilitator of inflammation by allowing the entry of immune cells and soluble inflammatory factors into the valvular tissue. Besides, vascular networks promote the weakening of valve tissue by changing the normal architecture (80, 81). A variety of growth factors can regulate angiogenesis, including vascular endothelial growth factor (VEGF)-A and MMPs that degrade fibrillar collagen or proteoglycan proteins allowing endothelial cells sprouting vessels by migration (82). Thus, angiogenesis besides contributing to tissue remodeling also can compromise mechanical stability of extracellular matrix. For the first time, we shown that mitral valves from patients with RHVD show an abundant neovascular network characterized by accumulation of large immature vessels, which is lacking in CAVD tissue (Figures 4A,B).

Besides blood vessels, lymphatic vasculature network was also observed in heart valves from patients with end stage RHVD as detected by colocalization of lymphatic endothelial cell receptor (LYVE-1) and podoplanin well-known markers of lymphatic endothelial cells (83) (Figures 4C,D). These vessels play role in lymph transport, tissue interstitial fluid absorption and serve as an entrance point for immune cells, favorable for optimal tissue function. and homeostasis (84). Autopsy results of adult heart valves show that lymphatic vessels in pathologic conditions such as RHVD present in a high (83). It is likely that neolymphogenesis is beneficial in the early phases of disease to maintain tissue homeostasis, but if become uncontrolled during the disease progression, may lead to pathological maladaptation. Persistent local inflammation impairs lymphatic contraction causing altered fluid transport (85). In addition, accumulation of collagen and fibrogenic molecules produced by smooth muscle cells and fibroblasts into the perilymphatic space causes capillary fibrosis and impairs vascular function (86). Thus, although lymphatic vessels are accumulating in lesions in large numbers, they are not able to exert their function properly.

In addition to growth factors and cytokines, hormones also participate in regulating angiogenesis. Estradiol influences hyaluronic acid synthesis, a major ECM ligand for the LYVE-1, suggesting an indirect effect on lymphatic homeostasis (87, 88). Also, while estrogen is described to reduce cardiovascular risk in women, it has been reported as a risk factor for disorders of lymphatic vascular system. Recently it has been shown that estrogen receptor alpha (ERα) directly regulates lymphoangiogenic genes promoting lymphatic endothelial cell migration and sprouting (89). Thus, excessive neolymphoangiogenesis observed in rheumatic mitral valves obtained predominantly from female patients, could be induced by estrogen which in turn can aggravate vessel sprouting in association with proinflammatory milieu.

## Final Considerations

No therapies are available to prevent or treat RHVD. Antibiotic prophylaxis is given to prevent repetitive episodes of ARF, and potentially limit the disease progression to severe valve dysfunction. However, there is no robust evidence of efficacy of secondary antibiotic prophylaxis in preventing recurrences of ARF (9). Additionally, there is no treatment to alter the likelihood or the severity of RHVD after an episode of ARF (8). Corticosteroids or intravenous immunoglobulins were tested in clinical trials to reduce the risk of heart valve lesions in patients with ARF, however, little evidence of benefit was found (90). Since RHVD results from an abnormal immune response, therapies targeting immune system could be more effective to avert valvular damage. Therefore, more research is needed to find specific immune components associated with the RHVD pathogenesis that will provide a more precise and effective therapeutical interventions to treat this devastating condition.

## Author Contributions

All authors listed have made a substantial, direct and intellectual contribution to the work, and approved it for publication.

## Conflict of Interest

The authors declare that the research was conducted in the absence of any commercial or financial relationships that could be construed as a potential conflict of interest.
